# Statistical Time-Series Analysis of Interferometric Coherence from Sentinel-1 Sensors for Landslide Detection and Early Warning

**DOI:** 10.3390/s21206799

**Published:** 2021-10-13

**Authors:** Marios Tzouvaras

**Affiliations:** 1 Department of Civil Engineering and Geomatics, Cyprus University of Technology, Limassol 3036, Cyprus; marios.tzouvaras@cut.ac.cy; 2 ERATOSTHENES Centre of Excellence, Limassol 3036, Cyprus

**Keywords:** Copernicus, SAR, landslides, early warning, critical infrastructure resilience

## Abstract

Landslides are one of the most destructive natural hazards worldwide, affecting greatly built-up areas and critical infrastructure, causing loss of human lives, injuries, destruction of properties, and disturbance in everyday commute. Traditionally, landslides are monitored through time consuming and costly in situ geotechnical investigations and a wide range of conventional means, such as inclinometers and boreholes. Earth Observation and the exploitation of the freely available Copernicus datasets, and especially Sentinel-1 Synthetic Aperture Radar (SAR) images, can assist in the systematic monitoring of landslides, irrespective of weather conditions and time of day, overcoming the restrictions arising from in situ measurements. In the present study, a comprehensive statistical analysis of coherence obtained through processing of a time-series of Sentinel-1 SAR imagery was carried out to investigate and detect early indications of a landslide that took place in Cyprus on 15 February 2019. The application of the proposed methodology led to the detection of a sudden coherence loss prior to the landslide occurrence that can be used as input to Early Warning Systems, giving valuable on-time information about an upcoming landslide to emergency response authorities and the public, saving numerous lives. The statistical significance of the results was tested using Analysis of Variance (ANOVA) tests and two-tailed *t*-tests.

## 1. Introduction

Landslides are a major geohazard causing human losses and significantly affecting the economy worldwide. Catastrophic landslides are widely distributed throughout Europe, however, with a great concentration in mountainous areas. Over the period of 1995–2014, in the 27 European countries, 476 landslides occurred causing a total of 1370 deaths and 784 injuries [1]. The global total annual losses caused by landslides are about EUR 18 billion, i.e., 17% of the average annual natural disaster losses (EUR 110 billion). In Europe, the average annual economic loss is EUR 4.7 billion, with landslides being responsible for approximately 17% of all fatalities caused by natural hazards [1].

Landslides can be triggered by various geological, geomorphological, physical, and anthropogenic factors [2]. Cyprus is located on the Mediterranean fault zone with a long history of seismic activity, with the main land displacement events taking place being landslides, rock falls, and ground subsidence. In fact, there are many active landslides and slope instabilities in areas with steep topography, whereas soil erosion by water affects many areas [3]. Moreover, extreme weather conditions such as drought and heavy rainfall can lead to the amplification of the soil erosion processes. In Cyprus, the occurrence of landslides has impacted built-up areas and critical infrastructure causing loss of human lives, injuries, destruction of properties, the abandonment and relocation of entire settlements, closure of roads, bridges, and disturbances in everyday commutes [3].

Currently, landslides and geological suitability issues are monitored by the Cyprus Geological Survey Department (GSD) via comprehensive in situ geotechnical investigations using conventional means, such as inclinometers, exploration boreholes, and wells in areas with known geological stability issues, such as Pissouri, Armou, Letimbou, Pentalia, Petra tou Romiou, etc. [3]. These extensive geological and geotechnical field investigations were carried out to develop landslide susceptibility and ground suitability maps and assess the landslide triggering factors, failure mechanisms, and events [4,5]. Both maps, however, cover only areas with a history of landslides and geological stability issues. The data collection for the detection of landslides via conventional means is time and money consuming, and the data are point-based and rather limited spatially, which is insufficient for the large areas that are usually affected. There is a need to cover more areas, with the relevant information being continuously updated. For ground deformation events, the timely provision of information is vital.

Currently, there is no comprehensive overview of the landslides’ economic impact, mainly since landslides are often associated with other natural hazards. The economic impact for Germany is EUR 300 million/year; Spain: EUR 170 million/year; Sweden: EUR 8–15 million/year; Norway: EUR 6.5 million/year; and Italy: EUR 1 billion/year. Satellite-based landslide monitoring has led to savings of up to 10% of costs by 2020, i.e., EUR 470 million/year, by systematically monitoring vulnerable areas, leading to a significant reduction in damages to properties, infrastructure, human lives, and the environment [6].

Earth Observation (EO) has evolved as a powerful and non-invasive tool of investigation that allows us to identify, observe, and measure objects, or phenomena and to detect, map and evaluate residing risks, without direct contact [7]. Active sensors, such as Synthetic Aperture Radar (SAR), can acquire images and obtain measurements anytime day or night, regardless of the weather conditions [8]. These characteristics allow for the systematic monitoring of landslides and their impact on infrastructure throughout the year over large areas [9].

The free availability of data from Copernicus, such as Sentinel-1, and other contributing missions [10], and the integration of conventional techniques with EO techniques could allow for the implementation of a near-real-time Early Warning System (EWS) [11,12,13]. The temporal and spatial characteristics of the Copernicus missions, such as the 12-day repeat cycle of the Sentinel-1 sensors in a single pass, ascending or descending (six-day repeat cycle of the two-satellite constellation at equator) can be used for mapping rapid changes in the landscape. The spatial resolution of Interferometric Wide (IW) swath images for Level-1 Single Look Complex (SLC) products that are available for the Eastern Mediterranean region provides additional advantages compared to their predecessors [8].

Interferometric and Differential Interferometric Synthetic Aperture Radar (InSAR/DInSAR) are space-based techniques that can determine the Earth’s surface topography and its temporal changes over large areas, with Line-of-Sight (LoS) accuracy of millimeters [14]. They have a proven record on determining the Earth’s surface topography and its temporal changes over large areas and have been used successfully for monitoring land subsidence and uplift due to landslides, earthquakes, volcanic eruptions, and human activities in various environments [15,16,17,18]. 

However, the C-band wavelength and the medium spatial resolution of the Sentinel-1 satellites can lead to various limitations in the detection, monitoring, and impact assessment of landslides via InSAR techniques, due to their complexity and fast development [19,20,21,22]. This phenomenon is called temporal phase aliasing and affects the phase unwrapping step of DInSAR [21,23]. Any ground deformation that exceeds the threshold of λ/4, i.e., 1.39 cm for Sentinel-1, between two SAR acquisitions, and can be two, three, or n times greater than this value, can be underestimated as all these deformations produce similar observed phases [24,25].

In such cases, interferometric coherence has been successfully used via the Coherent Change Detection (CCD) method [26,27,28,29]. Coherence, being a statistical value, cannot provide quantitative information about ground displacement, however, the identification of changes in coherence through interferometric SAR processing can provide useful information for the rapid detection of natural hazards [30,31]. Moreover, the integration of different SAR interferometry techniques can overcome any obstacles by combining their respective advantages [32].

Gradual land movements are known to precede major landslides. These are often on a scale of millimeters and are difficult to be noticed by local observers but can be detected via satellites using numerous SAR images, so that even very small terrain displacements are detected. With the increasing availability of suitable radar data, various time-series analysis techniques have been developed that can quantitatively derive deformation rates with increasing improvements in the temporal resolution. A time-series analysis approach taking advantage the availability of a large set of data and the multi-pass nature of Sentinel-1 satellites, can lead to a more accurate estimation of atmospheric contribution and topographic phase component, and allows a reduction in error sources [33,34]. Indeed, attempts have been made on the interpretation of InSAR outputs derived from time series analysis of SAR images to derive valuable information that can be used easily by relevant authorities for landslide monitoring [35,36].

Moreover, several studies have been carried out for the design, development, and implementation of early warning systems, as well as real and/or near-real-time landslide monitoring systems. They are mainly designed to predict the short-term behavior of individual landslides on a regional [11,13,37,38,39], and in few cases, territorial or international scale [40]. Their results have been widely used by stakeholders, decision/policy makers, and the public, assisting in the adoption of preventive measures by relevant authorities, the timely evacuation of areas in danger, saving many lives, and reducing damage to property and critical infrastructure.

EWS currently use various combinations of meteorological data, in situ geological and geotechnical data for areas with known ground stability issues, landslide inventories, and other landslide contributing factors, such as soil moisture, slope, elevation, etc. More recently, satellite images were integrated in EWS, through the calculation of ground deformation rates obtained through interferometric SAR image processing [11,12,41,42,43], and Normalized Difference Vegetation Indices (NDVI) calculated from optical satellite images [44,45].

The main aim of the present study is to find early evidence for upcoming landslides, through a comprehensive statistical analysis of coherence data obtained through InSAR processing using a series of Sentinel-1 satellite images provided for free by the Copernicus Programme and the freely available open-source software Sentinel’s Application Platform (SNAP) developed by ESA. To this direction, a landslide that was triggered by heavy rainfall on 15 February 2019, in a suburban area next to the motorway connecting the cities of Limassol and Paphos, is studied. 

The area of study is presented in the next section. The materials and methods used are presented in Section 3 and the results from data processing and analysis are presented in Section 4. Lastly, the discussion of the results and the conclusions are presented in Section 5 and Section 6 respectively.

## 2. Case Study

**Figure 1:**
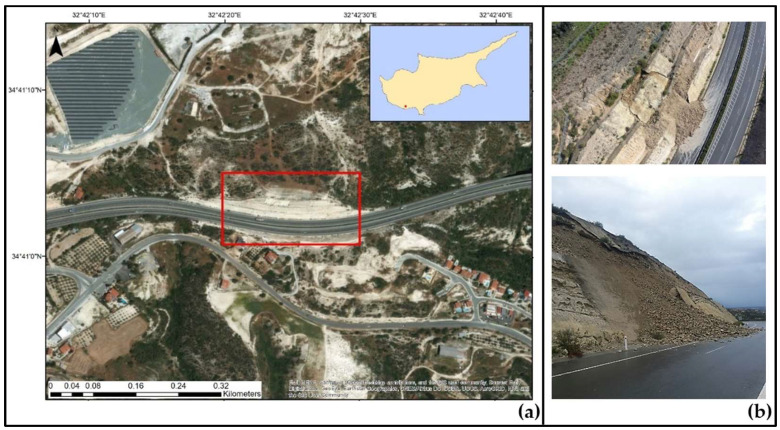
(**a**) The case study area by the A6 motorway near Pissouri where a landslide occurred on 15 February 2019. The wider area affected by the landslide is marked by a red rectangle [29]. (**b**) Photos from the landslide scene showing the impact of the landslide, provided by the Cyprus Geological Survey Department [29].

On 15 February 2019, after heavy precipitation, a landslide took place on an embankment of the A6 motorway connecting the cities of Limassol and Paphos, situated in Limassol District, near the village of Pissouri (Figure 1a). Tons of soil and rock from the embankment fell on the motorway (Figure 1b) causing great disturbance to thousands of commuters travelling on a daily basis from Paphos to Limassol. Traffic was diverted to the old Paphos–Limassol Road for approximately 35 days, until the completion of the rehabilitation works on 23 March 2019.

**Figure 2:**
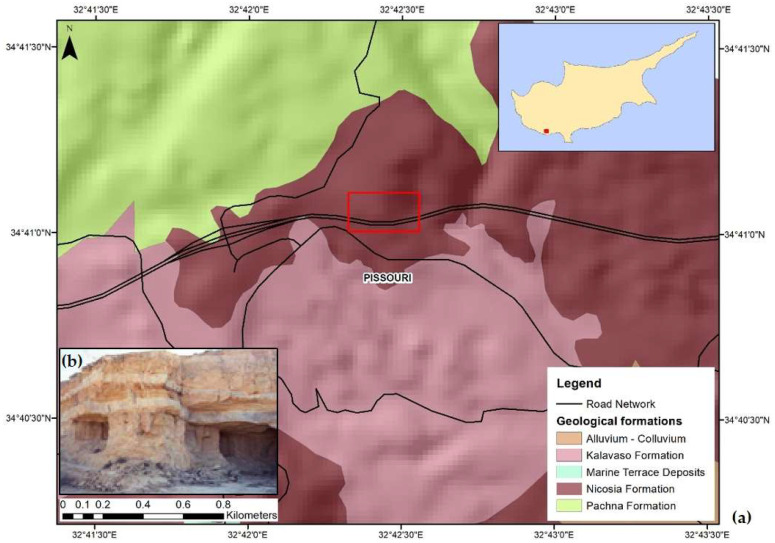
(**a**) The geological map and location of the study area [29] (Data provided by the Geological Survey Department). The wider area where the landslide occurred (marked in red) lies on (**b**) the Nicosia Formation [47].

The geology of the region (Figure 2a) consists of bentonite, lava, limestone, quartz sandstone, argillaceous shale and hornstone, serpentinite, pyroxenite, gabbro, chalks and marls, rocks with resistance to erosion and weathering [46]. The area that was affected by the landslide under study lies entirely on the Nicosia Formation (Figure 2b) that contains grey and yellow siltstones and layers of calcarenites and marls [47].

The main physical and geotechnical characteristics of the soil in the area were studied by the Geological Survey Department of Cyprus on 28 March–5 April 2016. A 30 m deep borehole investigation was carried out at the crown of the embankment [48], with the results listed in Table 1. The top 0.45 m are brown topsoil (sand gravel), and off-white subrounded conglomerates and pebbles, with presence of hephalogenous limestone at depths 0.5–0.6 m and 1.5–1.6 m, were found at depth 0.45–3.5 m. Grey-khaki sand was found at 3.5–3.9 m, followed by gravels and pebbles of sedimentary and ophiolitic origin until a depth of 4.5 m. Beige argyle was found until 4.8 m, and beige, soft, decomposed Marl with presence of orange oxidations was found at depth 4.8–7 m from the surface [48].

In the specific region, the most resistant rocks lie over very soft and fragmented masses, such as the Pissouri marl, that is susceptible to fast weathering [49]. The soils are particularly problematic and prone to landslides, with many landslides taking place in the last decade, mostly after extreme precipitation, affecting commuters significantly, due to their proximity to the road network [29].

More specifically, in January 2014, landslides and rockslides occurred in the areas adjacent to the case study area, towards the southwest, leading to the closure of approximately 1.5 km stretch of road in the direction from Paphos to Limassol for numerous days. Moreover, during the winter of 2014–2015, landslides and rockslides took place in the same area, that the landslide of January 2014 occurred, and in adjacent areas. Several parts of the old Limassol–Paphos Road and the A6 motorway connecting the two cities, remained closed because of mud and rocks on the road caused by a landslide. Soil removal works, slope normalization, and lining of stairs were carried out and traffic was diverted to alternative routes, causing significant disruption to the everyday commute.

## 3. Materials and Methods

Thirty-two (32) Sentinel-1 (1A and 1B) satellite images were downloaded from the Copernicus Open Access Hub [50], with the first image dated one month before the landslide 11 January 2019, and the last one nearly one month after the landslide on 12 April 2019. The south orientation of the embankment that was impacted by the landslide did not cause any limitations in the selection of a specific satellite pass direction, and as a result, both ascending and descending SAR images were downloaded.

All Sentinel-1 acquisitions (IW swath, Level-1, SLC with VV+VH polarization) were paired forming 28 interferometric SAR pairs that were used for the detailed monitoring of the development of the landslide. A 12-day temporal baseline was selected in all pairs, to minimize the impact that atmosphere and topography have on coherence results. Moreover, the suitability of all SAR image pairs was tested using a threshold of 200 m for the perpendicular baseline, which is normal for Sentinel-1 images, and 0.90 for the estimated modelled coherence. The interferometric SAR pairs, the acquisition platform, date, satellite pass direction, and other characteristics can be seen in Table 2, sorted in ascending order based on the master image acquisition date.

In total, 28 coherence maps were developed, based on the formed interferometric SAR image pairs, using the open-source software SNAP [51]. The coherence maps were stacked together, using geolocation, setting the coherence map of the first interferometric pair (11–23 January 2019) as the master image. The methodology for the development of coherence maps and the results are presented in detail in a previous publication of the corresponding author [29], and are not part of the present work, which concentrates on the statistical time-series analysis of the coherence results and the investigation of the potential extraction of early warning information for landslides using Copernicus datasets.

**Figure 3:**
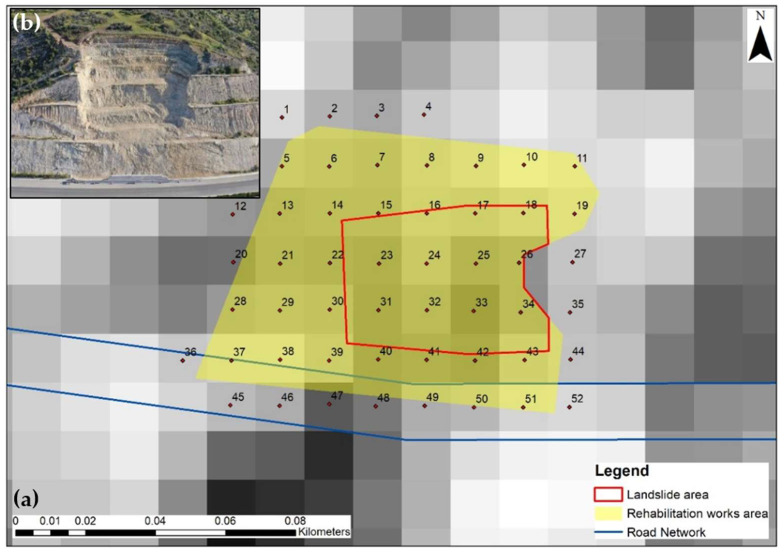
(**a**) Point grid used for the statistical analysis of the coherence values [29]. The red polygon outlines the extents of the area that was affected by landslide (AOI). The wider area of the rehabilitation works is marked in yellow and the A6 motorway is marked with blue line. (**b**) Site photo of the extent of the rehabilitation works at the embankment that was affected by the landslide [52].

ArcGIS was then used, to process the developed coherence maps, for further analysis of the results. For this purpose, as presented in Figure 3a, a 52-point grid was created following the outlines of the wider area affected by the landslide under study, which also covers the extents of the area of rehabilitation works (Figure 3b) marked in yellow. Following the directions of the Geological Survey Department and site visits, the precise extends of the landslide were defined, as presented by a red polygon in the following figure. This area will be referred to as Area of Interest (AOI) in the present study.

A thorough analysis was carried out based on the coherence values obtained in the two aforementioned areas. The coherence values obtained from Sentinel-1A and 1B images, for each pass direction, were studied separately, to investigate the performance of both satellites. Minimum, maximum, and average values were calculated for each satellite and pass direction, along with standard errors and standard deviations for each image pair. Finally, all results were tested statistically using Analysis of Variance (ANOVA) and two-tailed *t*-tests in Microsoft Excel.

## 4. Results

The results from the application of the proposed methodology are presented in detail in this section. The coherence values obtained from Sentinel-1A and 1B images, for each satellite pass direction, were studied separately for the wider area and the AOI. Although the statistical analysis of the coherence values is performed for the entire duration, the statistical test results are presented only for the pre-event period since this study is focused on the identification of early signs of upcoming landslides.

**Figure 4:**
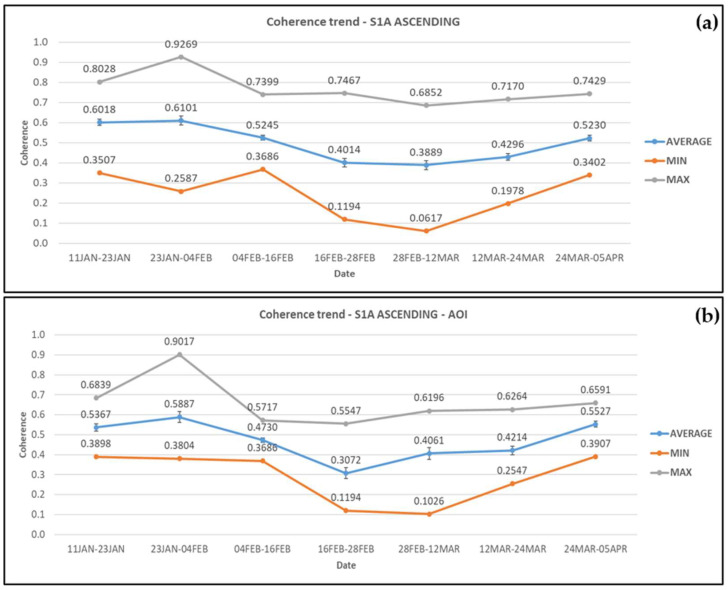
Coherence changes obtained from Sentinel-1A ascending satellite images for (**a**) the wider area and (**b**) the AOI. Error bars can be seen in the average coherence values.

From the coherence outputs of the ascending Sentinel-1A images, the average coherence seems stable in the greater area during 11 January–4 February 2019. The average coherence then dropped at the co-event pair, i.e., 4–16 February 2019, by 14%, and continued to decrease by 23.5% until the next pair. The coherence values during the period of rehabilitation works remained stable and then increased by 10.5% as the works completed and by 21.7% after the road re-opened. The average, minimum, and maximum coherence results are presented in Figure 4a below. 

Within the AOI, the coherence values decreased at a greater rate (19.7%) at the co-event SAR pair and at the start of the rehabilitation works (35%), reaching a minimum of 0.307. The coherence values were then increased by 36% as construction works reached completion and by 31.2% after the road opened to traffic. The average, minimum, and maximum coherence values for each SAR image pair are presented in Figure 4b. The peak that is seen in the maximum values at both cases, in the image pair dated 23 January–4 February 2019, is at point 43 which is located near the road.

**Figure 5:**
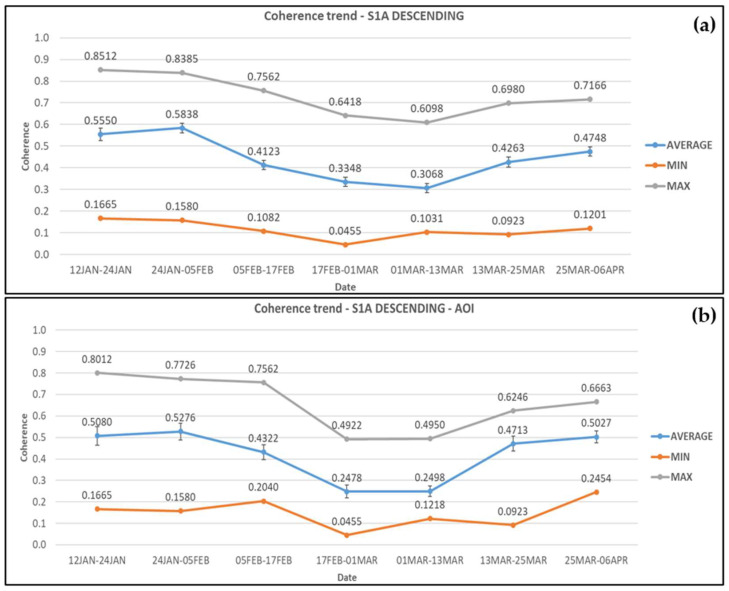
Coherence changes obtained from Sentinel-1A descending satellite images for (**a**) the wider area and (**b**) the AOI. Error bars can be seen in the average coherence values.

For Sentinel-1A descending, coherence values appear nearly stable on average before the landslide, i.e., 12 January–5 February 2019 in the wider area. There is a small increase of 0.028 (5.2%) on average that is though lower than the standard error estimated (±0.0286), and thus considered insignificant. Moreover, the coherence values dropped at the co-event pair (5–17 February 2019), by 29.4%, and continued to decrease by 18.8% until the next one (17 February–1 March 2019). During the period of rehabilitation works there was a small decrease of 8.4%, but the coherence values increased by 39% as the works ended and by 11.4% after the road re-opened to traffic. The average, minimum, and maximum coherence values obtained from each pair of images are presented in Figure 5a. 

Within the AOI, the coherence values from Sentinel-1A descending satellite images decreased by 18.1% at the co-event SAR pair and an additional 42.7% when the rehabilitation works commenced. The coherence values then increased by 88.7% as construction works reached completion and by 6.7% after the road re-opened to traffic. The average, minimum, and maximum coherence values obtained from each pair of images are presented in Figure 5b.

**Figure 6:**
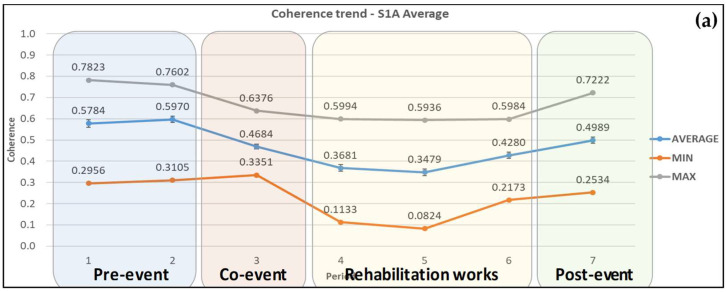

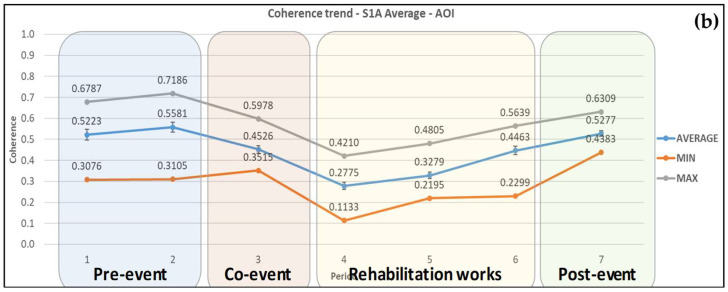
Average coherence changes obtained from Sentinel-1A ascending and descending satellite images for (**a**) the wider area and (**b**) the AOI. Error bars can be seen in the average coherence values.

By averaging the coherence pixel values from ascending and descending Sentinel-1A pairs, a clearer representation of the coherence changes trends in all three categories, i.e., minimum, maximum, and average values is provided, as shown in Figure 6a,b. At the same time, standard deviations were reduced leading to more reliable results. The horizontal axis values in the graphs were changed from dates to periods.

The peaks that existed in the maximum values in the case of the Sentinel-1A ascending images, and other small fluctuations evened out, resulting in a common trend in coherence changes in maximum, minimum, and average values. The average coherence is nearly stable within the error limits calculated in the pre-event period. It then decreased by 18.9% (co-event pair) and continued to decrease by 38.7% to reach the minimum value of 0.278 during the rehabilitation works. Then, the average coherence increased by 54.3% until the end of this period and by another 18.2% after the road opened. The maximum values of average coherence lie as expected within the pre-event and post-event periods.

**Figure 7:**
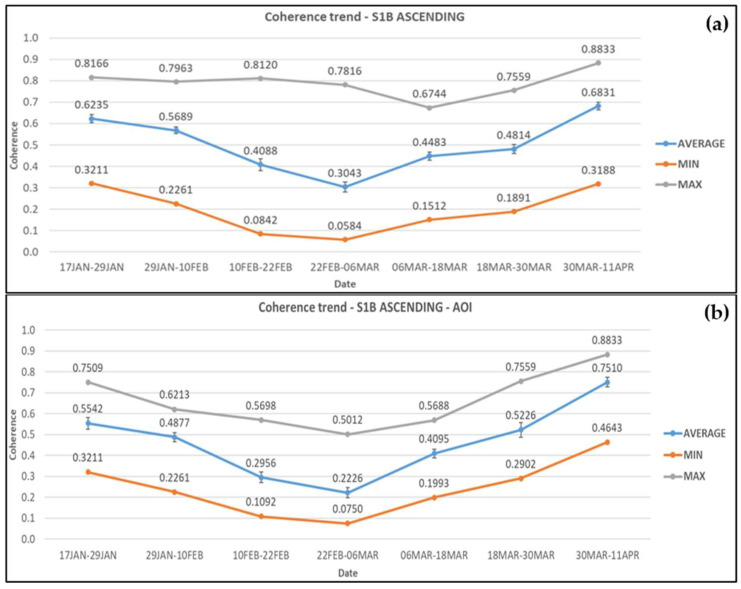
Coherence changes obtained from Sentinel-1B ascending satellite images for (**a**) the wider area and (**b**) the AOI. Error bars can be seen in the average coherence values.

In the case of Sentinel-1B ascending image pairs, in the wider area, coherence values started reducing on average by 8.8% before the landslide, i.e., 17 January–10 February 2019. Moreover, the coherence values continued to drop during the period 10–22 February 2019 (co-event pair) by 28.1%, and by 25.6% at the next pair dated 22 February–6 March 2019, reaching their lowest value on average (0.304). During the period of rehabilitation works there was a significant increase of 54.7% until their completion, and an additional increase of 41.9% after the road re-opened to traffic. The average, minimum, and maximum coherence values obtained at each period are presented in Figure 7a.

The coherence values decreased by 12% at the pre-event pair and further 39.4% at the co-event pair, within the AOI. The average coherence continued to drop by 24.7% during the start of rehabilitation works reaching its minimum value of 0.223. The coherence values then increased significantly by 111.6% as construction works reached completion and by 43.7% after the road re-opened to traffic. The average, minimum, and maximum coherence values obtained from each pair of images are presented in Figure 7b.

**Figure 8:**
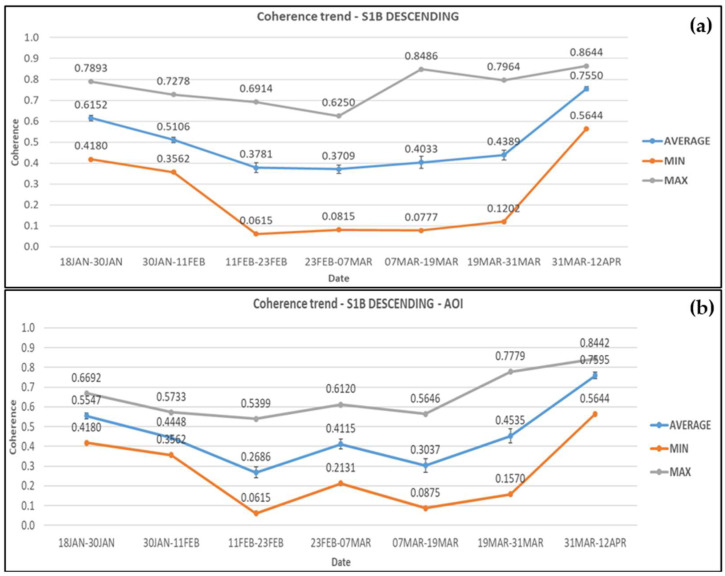
Coherence changes obtained from Sentinel-1B descending satellite images for (**a**) the wider area and (**b**) the AOI. Error bars can be seen in the average coherence values.

For Sentinel-1B descending, within the wider area, coherence values decreased by 17% on average before the landslide (18 January–11 February 2019) took place. The coherence values continued to drop during the period 11–23 February 2019 (co-event pair) by 26% reaching their minimum value of 0.371 on average. During the period of rehabilitation works there was an increase of 17.5% in coherence until their completion. Finally, the average coherence rose by 72% after the road re-opened to traffic. The average, minimum, and maximum coherence values obtained at each period are presented in Figure 8a. There is a peak at the maximum coherence (0.849) at the pair of SAR images dated 7–19 March 2019, which corresponds to point 35. This is due to the fact the specific point is located at the eastern boundaries of the area, and only a small portion of the pixel is within its limits.

Within the AOI, the coherence values from Sentinel-1B descending satellite images decreased by 20% at the pre-event pair and a further 39.6% at the co-event pair reaching its minimum average value of 0.269. During the rehabilitation works average coherence fluctuated, increasing initially by 53.2% and then decreasing by 26.2%. Average coherence then increased by 49.3% close to the completion of the works and continued increasing by 67.5% after the road opened to traffic reaching its maximum value of 0.76. The average, minimum, and maximum coherence values obtained from each pair of images are presented in Figure 8b.

Coherence values from Sentinel-1B ascending images appeared to be the best fit of what was expected based on the landslide occurrence, rehabilitation works, and road opening. Sentinel-1B descending also performed quite well except for a high peak in the maximum coherence values in the wider area for the pair 7–19 March 2019 and a high peak in maximum, minimum, and average coherence values within the AOI for the image pair 23 February–7 March 2019. Moreover, the results from Sentinel-1B ascending and descending satellite images show that there was a significant coherence loss, providing early warning information for the upcoming landslide, five days before the landslide occurred. The significance of this coherence drop is discussed further on. 

**Figure 9:**
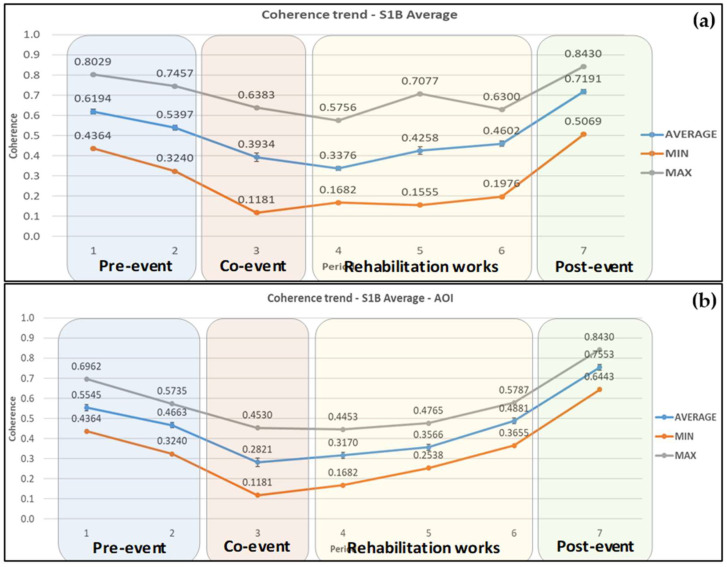
Average coherence changes obtained from Sentinel-1B ascending and descending satellite images for (**a**) the wider area and (**b**) the AOI. Error bars can be seen in the average coherence values.

By averaging the coherence pixel values from ascending and descending Sentinel-1B pairs, a clearer representation of the coherence changes trends in all 3 categories, i.e., minimum, maximum, and average values is provided, as shown in Figure 9a,b.

The high peak that existed in the maximum values for the wider area, but also for the minimum, maximum, and average values in the case of the Sentinel-1B descending images in the AOI, evened out completely, and there was a common trend in coherence changes in maximum, minimum, and average values. The minimum values, as shown in Figure 9a, lie within the rehabilitation works period and the maximum values in the post-event one. Through the averaging of coherence values, the standard deviations and standard errors of the samples were minimized. However, there was still a peak at the maximum coherence (0.708) during the period of rehabilitation works, which corresponds to point 27. This is due to the fact the specific point is located at the eastern boundaries of the area, and only a small portion of the pixel lies within its limits.

In the AOI, as presented in Figure 9b, minimum, average, and maximum coherence values follow the same trend, with maximum values being in the post-event period and minimum values in the co-event period. The range between minimum and maximum values is smaller compared with the previous data. The average coherence decreased by 15.9% during the pre-event period, giving valuable information for the evolution of the landslide. It continued decreasing more rapidly by 39.5% at the co-event period, reaching its minimum value of 0.282. Then, the average coherence increased by cumulatively 60% until the end of the rehabilitation works and then increased by another 54.7% after the road opening back again reaching its maximum value of 0.755.

For the AOI, within the boundaries of landslide alone, from the analysis of Sentinel-1A data, the minimum values of the average, maximum, and minimum coherence lie within the rehabilitation works period. In the case of Sentinel-1B data, there seems to be a better match of the data with the timeline of incidents, as all minimum values of the average, maximum, and minimum coherence, which were obtained from the analysis of the co-event ascending and descending pairs for the area of interest, were within the co-event period, i.e., the period that the landslide took place. In both cases the maximum values are all within the pre-event and post-event periods.

**Figure 10:**
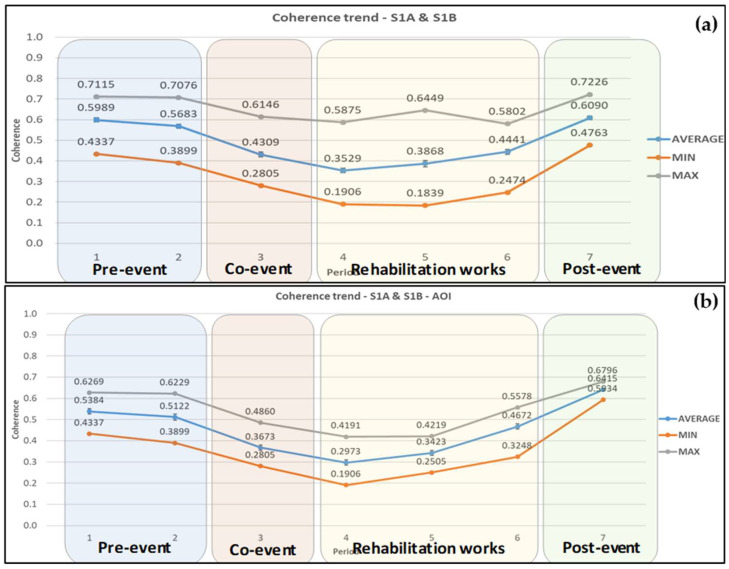
Average coherence changes obtained from Sentinel-1A and 1B ascending and descending satellite images for (**a**) the wider area and (**b**) the AOI. Error bars can be seen in the average coherence values.

The above results were combined further, as all coherence values, irrespective of satellite and pass direction, were averaged to investigate if the information obtained from the overall average coherence changes, provided more valuable input in the study conducted (Figure 10). In the wider area (Figure 10a), average maximum and minimum coherence values followed the same trend, having their minimum values during the period of rehabilitation works and their maximum during the pre-event and post-event periods. In the pre-event period, there was a coherence loss of 5.1% (0.599 to 0.568), and the coherence then dropped by 24.2% during the co-event period, and continued to decrease by 18.1% reaching its minimum value of 0.353 during the rehabilitation works period. Then average coherence increased by 26.4% in total by the end of the same period and by an additional 37.1% after the road opening, reaching its maximum value of 0.61. A high peak appears in the maximum coherence values in the middle of the period of rehabilitation works. By checking the point grid, this value is at point 11, located at the far north-eastern part of the wider area, near its boundaries. The fact that the entire pixel is not within the area of study introduced this unexpected rise in maximum values.

In the case of the AOI (Figure 10b), all three components, minimum, maximum, and average coherence followed the same trend with a small range of values between min and max. However, a noteworthy point is that their maximum values, within the post-event period, ranged only from 0.593 to 0.68. In the pre-event period, there was a coherence drop of 4.9%, i.e., from 0.538 to 0.512. The coherence then dropped by 28.3% during the co-event period, and continued to decrease by 19.1% reaching its minimum value of 0.297 during the rehabilitation works period. Then the average coherence increased by 51.6% in total by the end of the same period and by an additional 37.3% after the road opening, reaching its maximum value of 0.642.

Single factor Analysis of Variance (ANOVA) statistical tests and two-tail *t*-tests were carried out to prove the statistical significance of the results using a 95% level of confidence (*a* = 0.05) for all coherence results. In the present study, the potential of detecting signs of land displacement through Sentinel-1 images in the pre-event period, i.e., prior to the catastrophic landslide, were investigated.

At first, single factor ANOVA tests for all satellites and pass directions combinations were carried out, to prove that the samples’ means are not equal. Coherence values from three different samples were used, two from the pre-event and one from the co-event period. A null hypothesis that all samples’ means were equal was used, and the values of F and the critical values of *F* (*F_critical_*) determined from tables were compared. *F_critical_* is a function of the degrees of freedom of the numerator and the denominator and the level of significance. If *F* ≥ *F_critical_*, the null hypothesis is rejected, and the samples are significantly different. Coherence values were tested, with all results passing the ANOVA tests, except for results obtained from Sentinel-1A descending image pairs within the AOI (Table 3). This is also supported by the *p*-values, which are lower than the value of *a* set by the 95% level of confidence, providing strong evidence that the null hypothesis is invalid in all cases, apart from the results obtained from Sentinel-1A descending image pairs within the AOI. 

Since ANOVA tests cannot specify exactly where the difference lies, two-tailed *t*-tests were carried out to test further and determine if there was a significant difference between the means of the two samples, i.e., the two consecutive pre-event SAR image pairs, based on a null hypothesis that the means of two populations are equal. Therefore, t statistical and critical values (*t_critical_*) were calculated along with *p*-values. If *t* < *−t_critical_* or *t* > *t_critical_*, the null hypothesis is rejected. Moreover, *p* < *a* proves that the two samples differ significantly. The t-tests are expected to determine if the coherence changes are significant, and thus provide early warning indications for the landslide under study that occurred on 15 February 2019.

In both areas, i.e., the wider landslide area and the AOI, the coherence results from Sentinel-1A ascending and descending satellite images, and the average Sentinel-1A coherence values failed to reject the null hypothesis, and thus the datasets for the two consecutive pre-event image pairs do not differ significantly. Moreover, *p*-values, were greater than 0.05 (*a*), and failed to reject the null hypothesis. On the other hand, all results from Sentinel-1B ascending, descending and Sentinel-1B average, rejected the null hypothesis, with all t values calculated being outside the predefined limits set by the level of confidence, a finding that is also supported by the *p*-values that in all cases were lower than *a*. Moreover, based on the calculated t and *p* values for the overall average coherence results from both satellites and pass directions, the observed coherence losses are considered significant. The t-test results are presented in Table 4 below.

All results obtained from the ANOVA and t-tests are well supported from the findings of the comprehensive statistical analysis that was carried out in the present section. The observed variations between the average coherence values between the two consecutive Sentinel-1A (ascending, descending and average) image pairs were not great enough to assume that the results differ significantly. Therefore, the observed slight increase in coherence is considered insignificant in all cases. However, in the case of Sentinel-1B based results, the coherence loss observed in the results from ascending, descending, and their average, is considered significant, as proven by the statistical tests, providing valuable information for the evolution and occurrence of the landslide. Finally, the averaging of all coherence values also provided valuable results, with coherence values dropping before the occurrence of the landslide, a coherence difference that was found to be significant from the statistical tests carried out.

## 5. Discussion

Based on the findings that were presented in the previous section, both Sentinel-1A and Sentinel-1B satellites performed quite well in landslide detection. The sole difference is in the pre-event period where images from Sentinel-1A showed a small increase in average coherence values, in comparison with a more significant reduction in average coherence values in the case of Sentinel-1B images during the same period. The significance of the observed difference was proved through the ANOVA and t-tests, and the respective *p*-values. As all the acquisition and sensor characteristics of the Sentinel-1A and Sentinel-1B satellites are nearly identical, an explanation to this could be the difference of the SAR image acquisition dates of the Sentinel-1A and Sentinel-1B. Sentinel-1A images were acquired 10–11 days, whereas the Sentinel-1B images 4–5 days before the landslide. 

Indeed, as was observed during the comprehensive statistical analysis, the coherence loss was greater as we approached the date of the landslide occurrence. Moreover, the difference in the acquisition dates could have introduced additional phase decorrelations due to the residing meteorological conditions on the specific dates. In all cases, the maximum values can be seen during the pre-event and post-event periods, and the lowest either in the co-event or the rehabilitation works period. Overall, minimum, maximum, and average coherence values tended to follow the same pattern over the entire period of study, with this observation being intensified in the coherence values calculated by averaging coherence results obtained from all SAR pairs in the AOI (Figure 10b). Nevertheless, the range of values was reduced.

In some cases, pixels that were not fully included in the area under study provided unexpected coherence results. However, this was minimized through the averaging of the results from Sentinel-1A and 1B satellites in both pass directions. In fact, odd coherence variations, such as peaks or troughs, were minimized in the average coherence changes. 

Also noteworthy is the fact that there was a far more significant coherence change within the AOI than in the wider area under study, showing the impact that the landslide had on the specific area. Indeed, the minimum, average, and maximum coherence results were primarily lower with the AOI compared to the results in the wider area. The specific finding strengthens the case that the coherence loss observed in AOI was due to the upcoming landslide, as the residing geological and meteorological conditions were identical in both areas. These are all justified by the comprehensive statistical analysis that was conducted (Figure 4, Figure 5, Figure 6, Figure 7, Figure 8, Figure 9 and Figure 10) and by the statistical tests performed (Table 2 and Table 3). 

## 6. Conclusions

The Copernicus Programme and the Sentinels, with their enhanced temporal and spatial characteristics provide opportunities for the systematic monitoring and impact assessment of landslides and other natural hazards. The information extracted can be exploited by numerous stakeholders and relevant authorities on disaster/emergency management sector, and raise awareness to the public for upcoming landslides, protecting the environment and saving lives.

Apart from the analysis of phase for the calculation of ground deformation rates through InSAR image processing, interferometric SAR coherence can be used to develop quick products, as processing time is minimum. This, in turn, can facilitate the development of Early Warning Systems (EWS), as the entire process, including data acquisition, image processing, and the release of the warning for upcoming dangers must be minimized to provide real and/or near-real-time landslide monitoring.

Compared to conventional in situ landslide monitoring methods, the proposed methodology overcomes accessibility, coverage, and meteorological conditions obstacles, through the use of SAR satellite images, exploiting their temporal, spatial, and multi-pass characteristics, and all-weather acquisition capabilities. Moreover, the proposed methodology is advantageous compared to other EO based landslide monitoring methodologies, providing valuable information on landslide imminent danger on time, through rapid satellite image processing, and a statistical analysis of interferometric coherence, as discussed earlier.

Indeed, the present study proved that the exploitation of the datasets provided freely by the Copernicus Programme, can indeed provide valuable information, not only for impact assessment but also for the early identification and detection of landslide. Results from Sentinel-1B satellite provided indication of early warning in the case of the landslide under study, as information of significant coherence loss was given five days prior to the landslide occurrence. Similar indications were observed after averaging the coherence values from both satellites and pass directions. The coherence losses observed were considered significant based on the ANOVA and two-tailed t-tests carried out between the pre-vent SAR image pairs.

A step forward in the development and implementation of an operational EWS, would be the use of optical and radar satellite data of different characteristics from other sources (COSMO-SkyMed, TerraSAR-X, WorldView, GeoEye, etc.), and their fusion if needed, to obtain data continuously for systematic extraction of information. Moreover, the automation of the proposed methodology for data acquisition, processing, and the release of valuable information on time is suggested to protect the environment and save lives that are in danger.

## Figures and Tables

**Table 1:** Main soil physical and geotechnical characteristics at the area affected by the landslide. Data obtained from borehole investigation carried out by the Geological Survey Department [48].

Geological Description	Depth (m)	Gradation (%)	Moisture Content (w)	Bulk Density (KN/m^3^)	Dry Density (KN/m^3^)
Khaki, hard, weak, moderately decomposed Marl with presence of orange oxidations	9.00	Sand: 34% Silt: 51% Clay: 15%	25.68	19.89	15.83
	11.50	Sand: 21% Silt: 61% Clay: 18%	34.35	19.09	14.21
	15.00	-	-	20.09	14.98
	18.90	-	-	18.50	13.22
Grey, hard, relatively healthy Marl	20.50	-	-	18.76	13.96
	23.50	Sand: 19% Silt: 60% Clay: 21%	37.23	19.30	14.07
	30.00	Sand: 4% Silt: 47% Clay: 49%	40.56	18.89	13.44

**Table 2:** SAR image interferometric pairs [29]. Image pairs 1–8 are in the pre-event, 9–12 are in the co-event, and 13–28 are in the post-event period.

No.	Platform	Date (Master)	Date (Slave)	Pass Direction	Perpendicular Baseline	Modelled Coherence
1	Sentinel-1A	11/01/2019	23/01/2019	Ascending	16.36 m	0.98
2	Sentinel-1A	12/01/2019	24/01/2019	Descending	108.39 m	0.90
3	Sentinel-1B	17/01/2019	29/01/2019	Ascending	43.40 m	0.95
4	Sentinel-1B	18/01/2019	30/01/2019	Descending	34.79 m	0.96
5	Sentinel-1A	23/01/2019	04/02/2019	Ascending	155.91 m	0.86
6	Sentinel-1A	24/01/2019	05/02/2019	Descending	79.26 m	0.92
7	Sentinel-1B	29/01/2019	10/02/2019	Ascending	23.22 m	0.97
8	Sentinel-1B	30/01/2019	11/02/2019	Descending	46.48 m	0.95
9	Sentinel-1A	04/02/2019	16/02/2019	Ascending	102.44 m	0.90
10	Sentinel-1A	05/02/2019	17/02/2019	Descending	12.46 m	0.98
11	Sentinel-1B	10/02/2019	22/02/2019	Ascending	17.44 m	0.97
12	Sentinel-1B	11/02/2019	23/02/2019	Descending	86.63 m	0.92
13	Sentinel-1A	16/02/2019	28/02/2019	Ascending	84.72 m	0.92
14	Sentinel-1A	17/02/2019	01/03/2019	Descending	14.88 m	0.97
15	Sentinel-1B	22/02/2019	06/03/2019	Ascending	63.25 m	0.94
16	Sentinel-1B	23/02/2019	07/03/2019	Descending	10.12 m	0.98
17	Sentinel-1A	28/02/2019	12/03/2019	Ascending	3.15 m	0.99
18	Sentinel-1A	01/03/2019	13/03/2019	Descending	87.86 m	0.92
19	Sentinel-1B	06/03/2019	18/03/2019	Ascending	30.08 m	0.96
20	Sentinel-1B	07/03/2019	19/03/2019	Descending	75.78 m	0.93
21	Sentinel-1A	12/03/2019	24/03/2019	Ascending	17.67 m	0.97
22	Sentinel-1A	13/03/2019	25/03/2019	Descending	63.94 m	0.93
23	Sentinel-1B	18/03/2019	30/03/2019	Ascending	82.29 m	0.92
24	Sentinel-1B	19/03/2019	31/03/2019	Descending	9.54 m	0.98
25	Sentinel-1A	24/03/2019	05/04/2019	Ascending	51.98 m	0.94
26	Sentinel-1A	25/03/2019	06/04/2019	Descending	57.14 m	0.94
27	Sentinel-1B	30/03/2019	11/04/2019	Ascending	30.86 m	0.96
28	Sentinel-1B	31/03/2019	12/04/2019	Descending	24.92 m	0.97

**Table 3:** ANOVA test results for coherence values from Sentinel-1A, Sentinel-1B in ascending and descending pass direction, average Sentinel-1A, average Sentinel-1B, and overall Sentinel-1A and Sentinel-1B average. The successful tests are in green, whereas those that failed are in red.

Platform	Pass Direction	Wider Area			Area of Interest (AOI)		
		*F*	*F_critical_*	*p*	*F*	*F_critical_*	*p*
Sentinel-1A	ascending	7.5352	3.0552	0.0008	8.3802	3.1588	0.0006
Sentinel-1A	descending	14.4506		1.78 × 10^−6^	1.6403		0.2029
Sentinel-1A	AVERAGE	22.1747		3.49 × 10^−9^	5.4171		0.0070
Sentinel-1B	ascending	28.5609	3.0552	2.88 × 10^−11^	28.8219	3.1588	2.24 × 10^−9^
Sentinel-1B	descending	50.0277		1.92 × 10^−17^	57.5974		2.07 × 10^−14^
Sentinel-1B	AVERAGE	53.3067		2.71 × 10^−18^	68.3903		7.15 × 10^−16^
S1A and S1B	AVERAGE	65.3343	3.0552	3.08 × 10^−21^	46.6924	3.1588	9.82 × 10^−13^

**Table 4:** t-test results for coherence values from Sentinel-1A, Sentinel-1B in ascending and descending pass direction, and average Sentinel-1A and Sentinel-1B coherence values. The successful tests are in green, whereas those that failed are in red.

Platform	Pass Direction	Date (Master)	Date (Slave)	Wider Area			Area of Interest (AOI)		
				*t*	*t_critical_*	*p*	*t*	*t_critical_*	*p*
Sentinel-1A	ascending	11/1-23/1/19	23/1-4/2/19	−0.305	2.008	0.7617	−1.574	2.093	0.1319
Sentinel-1A	descending	12/1-24/1/19	24/1-5/2/19	−1.623		0.1108	−0.846		0.4079
Sentinel-1A	AVERAGE	Pre-event 1	Pre-event 2	−1.157		0.2528	−2.062		0.0531
Sentinel-1B	ascending	17/1-29/1/19	29/1-10/2/19	3.772	2.008	0.0004	2.240	2.093	0.0372
Sentinel-1B	descending	18/1-30/1/19	30/1-11/2/19	10.544		2.05 × 10^−14^	10.583		2.10 × 10^−9^
Sentinel-1B	AVERAGE	Pre-event 1	Pre-event 2	11.274		1.85 × 10^−15^	6.691		2.14 × 10^−6^
S1A and S1B	AVERAGE	Pre-event 1	Pre-event 2	3.756	2.008	0.0004	3.284	2.093	0.0039

## Data Availability

All background information used in this article can be found in the appropriated references, while the satellite images used are freely accessible from the Copernicus Open Access Hub.
